# Separation and Identification of Highly Fluorescent Compounds Derived from *trans-*Resveratrol in the Leaves of *Vitis vinifera* Infected by *Plasmopara viticola*

**DOI:** 10.3390/molecules17032773

**Published:** 2012-03-06

**Authors:** Jan Tříska, Naděžda Vrchotová, Julie Olejníčková, Rudolf Jílek, Radek Sotolář

**Affiliations:** 1 Laboratory of Metabolomics and Isotopic Analyses, Global Change Research Center, Academy of Sciences of the Czech Republic, Branišovská 31, České Budějovice 370 05, Czech Republic; Email: vrchotova.n@czechglobe.cz (N.V.); jilek.r@czechglobe.cz (R.J.); 2 Laboratory of Plant Ecological Physiology, Global Change Research Center, Academy of Sciences of the Czech Republic, Zámek 136, Nové Hrady 373 33, Czech Republic; Email: olejnickova.j@czechglobe.cz; 3 Department of Viticulture, Faculty of Horticulture, Mendel University in Brno, Valtická 337, Lednice 691 44, Czech Republic; Email: radek.sotolar@mendelu.cz

**Keywords:** *trans*-resveratrol, transformation, 2,4,6-trihydroxyphenanthrene-2-*O*-glucoside, *Plasmopara viticola*

## Abstract

A method for identification of highly fluorescent compounds in vine leaves infected by *Plasmopara viticola* was developed using reversed phase liquid chromatography with simultaneous diode array and fluorometric detection. Fluorescent compounds were extracted from leaves with a methanol-water mixture (70:30). Separation by HPLC was performed using a C_18_ column and gradient elution with water-acetonitrile mixtures (20–80% of acetonitrile). The main unknown fluorescent compound was identified by line spectral comparison with a standard obtained by UV photoisomerization of *trans-*resveratrol glucoside, and its structure was confirmed by liquid chromatography-mass spectrometry. Identification and structural elucidation of the fluorescent compound in the leaves of *Vitis vinifera* allows early detection of *Plasmopara viticola* invasion.

## 1. Introduction

**Figure 1:**
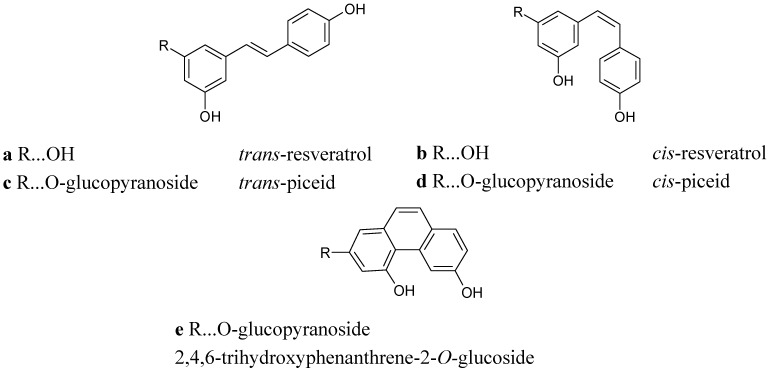
Chemical structures of the stilbene and phenanthrene derivatives.

Hydroxylated stilbenes are phytoalexins synthesized by plants, especially in the fruit skins, leaves, and roots, in response to fungal infections and UV light [1,2,3]. Resveratrol (stilbene-3,5,4'-triol) is a constituent of vine plants and wine that has attracted enormous research interest in recent years due to its broad range of biological activities, especially its antioxidant activity. Resveratrol is known to occur in wine in free and glycosidically bound forms. Free *trans*- and *cis*-resveratrol (Figure 1a,b) are present in concentration ranges of 0.2–13 mg/L in red wines and 0.1–0.8 mg/L in white wines. For the bound forms of resveratrol, concentrations of the so-called piceids (*trans*- and *cis*-resveratrol-3-*O*-β-glucosides) (Figure 1c,d), are reported to be in a ranges of 0.3–9 mg/L in red and 0.1–2.2 mg/L in white wines [4,5,6]. Ribeiro de Lima *et al.* [7] determined piceids in Portuguese red wines even in concentrations up to 68 mg/L. Large amounts of *cis*-and *trans*-piceid accumulate in healthy berries of different grape varieties [8], as well as following UV light treatment [9] or powdery mildew (*Uncinula necator*) infection [10]. Interaction between *Plasmopara viticola* infection and stilbene synthesis in leaves and berries of ten Cabernet Sauvignon clones were recently studied by van Zeller *et al.* [11]. The isolation and structural characterization of 10 major viniferins in the leaves infected by *Plasmopara viticola* was very recently described by Mattivi *et al*. [12]. The literature includes numerous references to content of phytoalexins, and especially *trans-*resveratrol, in grapevines and wines, but only a few references mention the content of that compound in the leaves, e.g., the first original paper from Langcake and Pryce [1] and more recently that of Babíková *et al*. [13]. The *cis-* form of resveratrol is normally present to a lesser extent in grape leaves. The presence of *cis-*resveratrol in the leaves, berries and wines is, according to the literature, a consequence of biological procedures (so-called “bioproduction”) for treating *V. vinifera* plants in the vineyards [14]. This means that *cis-*resveratrol could be a “biomarker” for wines made from grapes not treated with any chemicals and which were under increased impact of “biochemicals” produced most probably by *Botrytis cinerea* or *P. viticola*.

The antioxidant activity of resveratrol has been linked to reduced mortality from coronary heart disease (CHD) [15]. Resveratrol also inhibits human platelet aggregation *in vitro* [16] and modulates eicosanoid synthesis toward a pattern likely to be protective against CHD [17,18]. More recently, Gehm *et al*. [19] have reported estrogenic properties of resveratrol that may also contribute to the reported cardioprotective effects of wine consumption. The literature includes an increasing number of publications addressing various aspects of biological and health effects of resveratrol, e.g., recent review of Guilford and Pezzuto [20]. The urgent need to exchange experience in this area led to organization of the first International Conference of Resveratrol and Health, held in Copenhagen in 2010. All articles dealing with health aspects of resveratrol were published in a special issue of the Annals of the New York Academy of Sciences in 2011 [21].

*Vitis vinifera* varieties are highly susceptible to downy mildew (*P. viticola*) infection, and some fungicides are needed to sustain economic grape production. Fungicides, especially copper derivatives, are directly linked to ecological harm, however, and therefore reasonable alternative mechanisms for grapevine protection are urgently needed. *trans-*Resveratrol and its derivatives are main phytoalexins of *Vitis vinifera* plants. *trans-*Resveratrol is synthesized in the free form by the action of stilbene synthase and then is rapidly glycosylated by a glycosyltransferase to *trans-*resveratrol glucoside. Both free and glycosylated forms of *trans-*resveratrol could isomerize to *cis-* forms either by UV irradiation or in healthy plants most probably by a *cis-*isomerase, which has not been characterized to date [22]. Further isomerization to a trihydroxy phenanthrene derivative would be catalyzed by *cis-*isomerase, most probably present together with other enzymes in *P. viticola* tissues, and/or by the sun’s UV irradiation. The first step in the verification of this hypothesis is to find phenanthrene derivatives both in the healthy and infected grapevine leaves.

## 2. Results and Discussion

**Figure 2:**
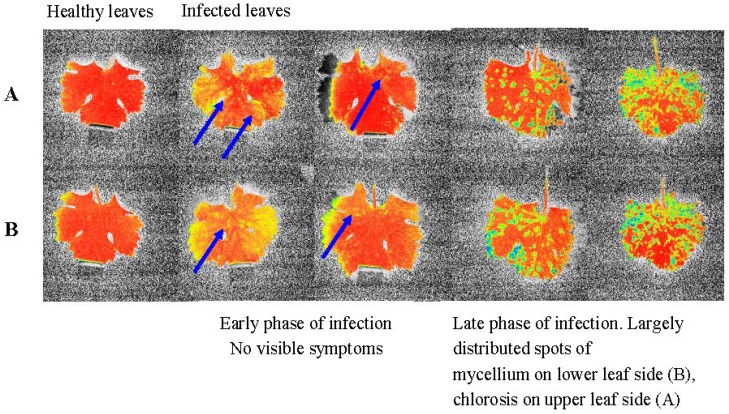
Infection by *Plasmopara viticola* progress in the leaves of susceptible variety Sauvignon. Upper row **A** illustrates increasing chlorosis on the upper side of the leaf, lower row **B** illustrates the growth of mycelium on the lower leaf side. The arrows highlight the spots where the infection (necrosis) started.

The distribution of fluorescence emission was measured in healthy and infected grape leaves during July 2009 and 2010 at vineyards in Kostice and Lednice, Czech Republic. These vineyards are located at an altitude of 176 m a.s.l and have absolutely no fungicidal protection (biodynamic). Controlled varieties were grafted on Teleki 5C rootstock. The training is central (trunk 0.7 m) with one cane (8 to 10 buds). The varieties are planted 1 m apart. The inter-row width is 2.2 m. Natural occurrence of fungi is an early and significant occurrence, but was an artificial infection of shrubs (mixture of water and spray-pathogen spores, loose-zoospore). From each variety (susceptible varieties: Chardonnay, Sauvignon, Muscatel, Müller Thurgau and Alibernet, resistant varieties: Cerason, Malverina, Hibernal and Laurot), 10 healthy leaves and 10 infected leaves in total were measured. In the case of sensitive varieties in the early stage of infection when no noticeable visible symptoms were yet visible, Φ_II_ showed heterogeneous distribution of small patches on the leaves having locally reduced photosynthetic activity due to infection. As the infection progressed, the number of places with reduced photosynthetic activity increased continuously and their total area was also increasing. In the case of resistant varieties, the places with reduced Φ_II_ at an early stage of infection were far more extensive. While in such cases there was no expansion of the affected areas, the plants localized the infection into a large spot on the leaf, which was followed by necrosis. Necrosis of infected tissue is clearly visible in Figure 2 for the Sauvignon variety. 

**Figure 3:**
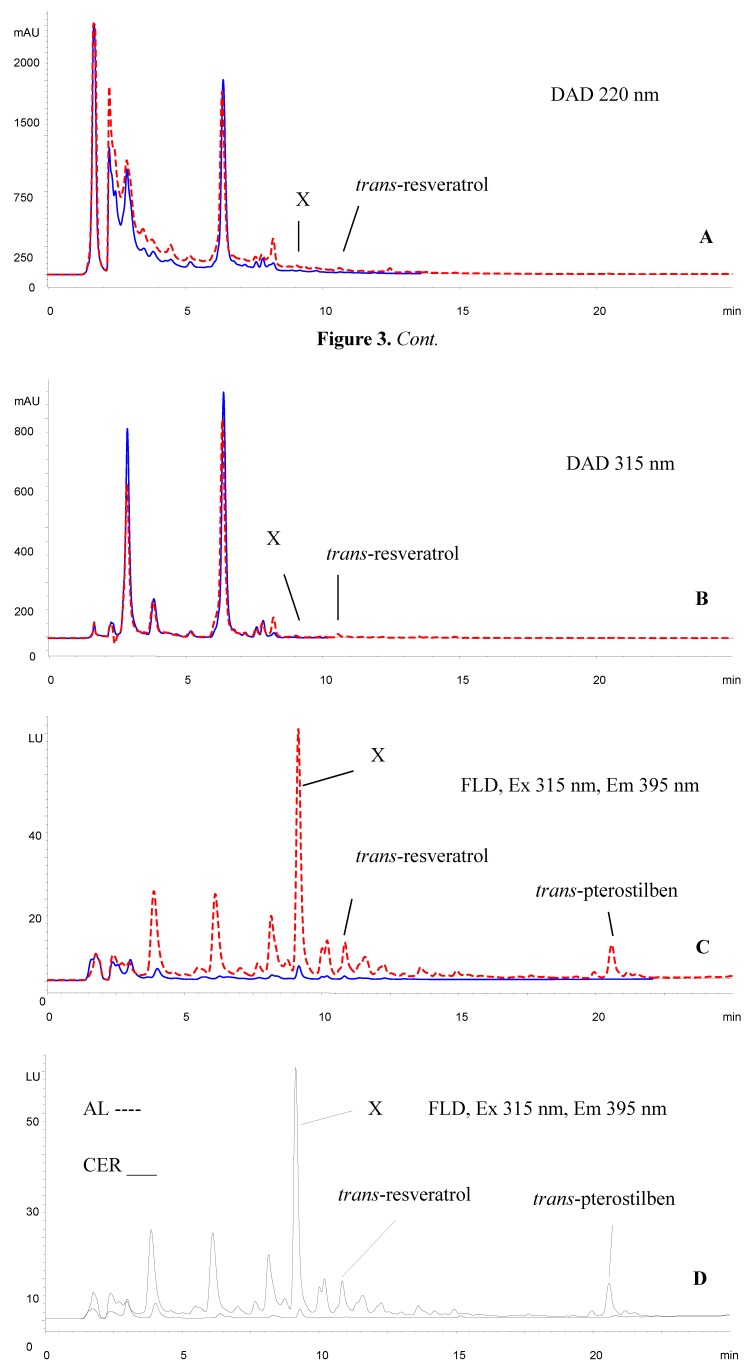
HPLC chromatograms of the extracts of the Alibernet variety (susceptible) leaves. Comparison of healthy leaves (blue line) and leaves infected by *Plasmopara viticola* (red dashed line). (**A**) Chromatogram of the extract at 220 nm. (**B**) The same chromatogram at 315 nm. (**C**) Chromatogram of the extract using FLD detector. Compound X is 2,4,6-trihydroxyphenanthrene-2-*O*-glucoside. (**D**) Comparison of the susceptible infected variety Alibernet (dashed line) and resistant infected variety Cerason (solid line).

For all leaves, significant Φ_II_ changes in leaf area were correlated with the infected sites. The samples for determining phenolic compounds and photosynthetic pigments were taken in the areas with reduced photosystem II activity (low Φ_II_). Methanolic extracts of the samples were measured by UV (DAD) and fluorescence detection, whereby four peaks were clearly visible in the retention times window starting at 4 min. The largest, most distinct peak had a retention time of 9.15 min (see Figure 3).

Formation of phenanthrene derivatives as final products of the UV photo isomerization reaction of *trans-*resveratrol to *cis*-resveratrol is described in the literature, whereby the latter undergoes a photo cyclization reaction to phenanthrene [23,24]. Without elucidating any structure for a new compound, Merás *et al*. have postulated that possible phenanthrene derivatives could occur as a result of UV irradiation of the piceid solution [25].

**Figure 4:**
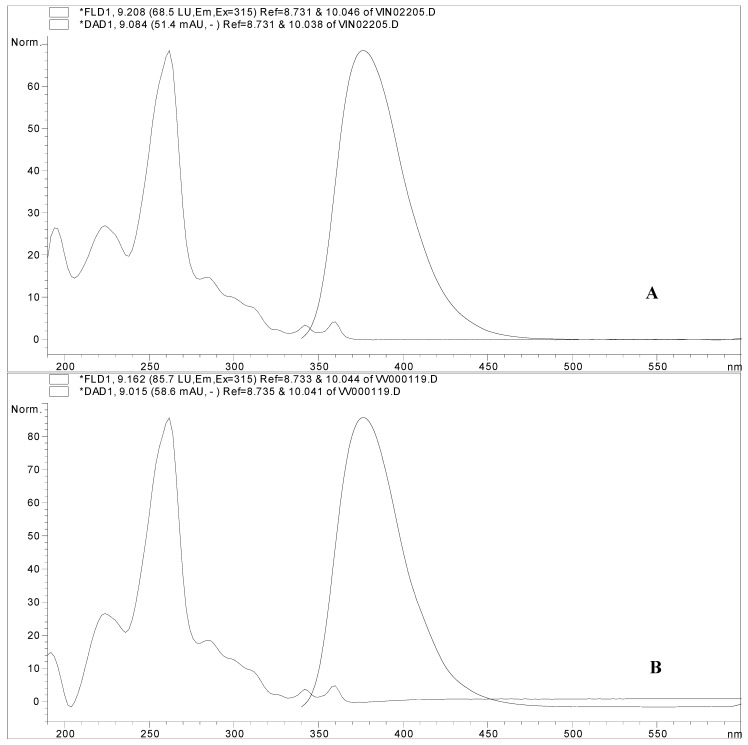
(**A**) UV spectrum of irradiated *trans-*piceid standard (dashed line) and its emission spectrum (solid line). (**B**) UV spectrum of the Alibernet variety leaves extract (dashed line) and its emission spectrum (solid line), which is peak X in the chromatogram shown in Figure 3.

To test the possibility of *trans*-piceid phototransformation into our highly fluorescent compound, an experiment was performed whereby *trans*-piceid was irradiated in an open vial at 254 nm for 4 h. After irradiation, there appeared in addition to the two already existing components (*trans*- and *cis*-piceid) a new component (x), which eluted also at the retention time of 9.15 min overlapping with the peak and UV spectra of the unknown substance (Figure 4). 

**Figure 5:**
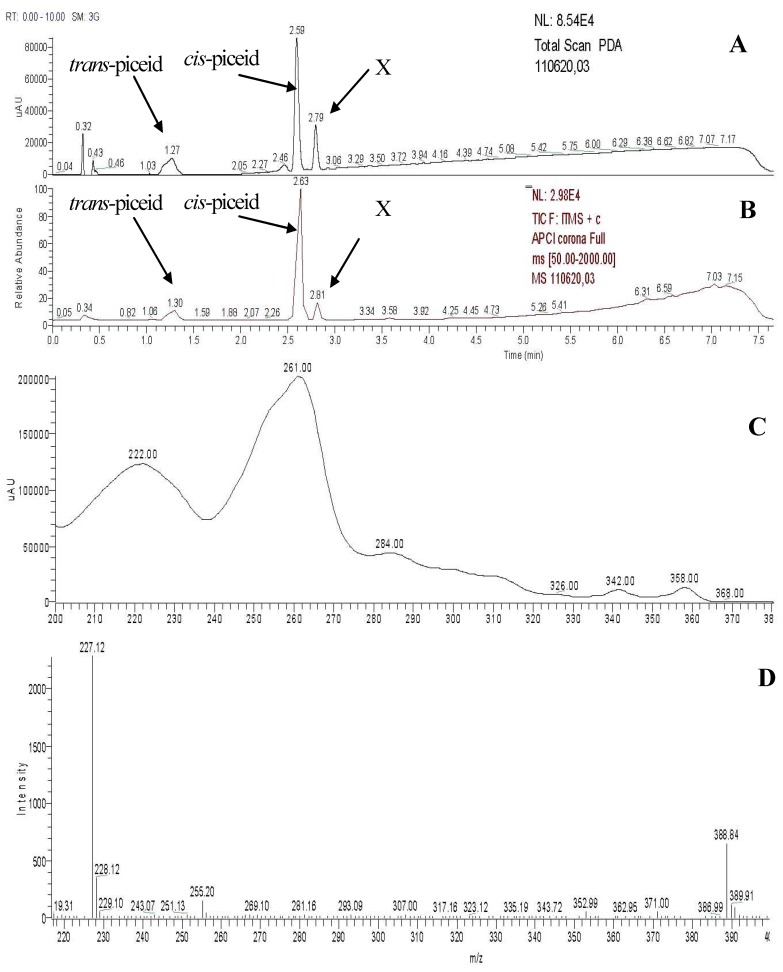
LC-MS analysis of the irradiated *trans-*piceid standard. (**A**) Total scan from the photo diode array detector. (**B**) Full spectrum from LC-MS APCI measurement in the positive mode. (**C**) UV spectrum of the peak with the retention time of 2.80 min. (**D**) Mass spectrum from LC-MS APCI measurement in the positive mode of the peak with the retention time of 2.80 min.

To confirm that this new peak is the assumed phenanthrene derivative, LC-MS measurements were carried out using the Accela Fleet ion trap LC-MS instrument and APCI ionization technique in the positive mode. By LC-MS measurement of the peak with the retention time 2.80 min, we observed two distinct ions: a molecular ion at *m/z* 389 [M+H]^+^ and an ion at [M–162]^+^ (Figure 5, Table 1). The fragment with *m/z* 162 is anhydroglucose and it is typical for the fragmentation of glucosides. There is ion 135^+^ missing in the fragmentation spectrum, which is typical for *trans-* or *cis*-resveratrol. In UV spectrum there is a maximum at 261 nm, which is typical for trihydroxy phenanthrene derivative—see Montsko *et al*. [24].

**Table 1:** MS/MS measurements of 2,4,6-trihydroxyphenanthrene-2-*O*-glucoside.

MS technique	Parent ion	Fragments ions
+ APCI	389^+^	371^+^ (M-H_2_O)
	389^+^	353^+^ (M-2H_2_O)
	389^+^	335^+^ (M-3H_2_O)
	389^+^	227^+^ (M-162)
	227^+^	209^+^ (M-H_2_O)
	227^+^	199^+^, 181^+^, 157^+^

This molecular ion and typical fragmentation provide convincing results, as the observed data and fragmentation pattern are consistent with the proposed structure. Therefore, according to all experimental data, our main unknown fluorescent compound must be 2,4,6-trihydroxyphenanthrene-2-*O*-glucoside (Figure 1e). This trihydroxyphenanthrene *O-*glucoside was recently obtained from a multilayer countercurrent chromatography fraction after careful purification using a series of separation steps [26]. As to the hypothetical formation of 2,4,6-trihydroxyphenanthrene-2-*O*-glucoside from *cis*-piceid, photochemical reactions as well as enzymatic activities can be considered likely. To the best of our knowledge, 2,4,6-trihydroxyphenanthrene-2-*O*-glucoside is reported here for the first time as a natural product whose amount is significantly increased in relation to the impact of *P. viticola.* The concentration of 2,4,6-trihydroxyphenanthrene-2-*O*-glucoside in the non-infected leaves is in the range of 2.36–14.24 μg.g^−1^ f.w. for susceptible varieties and in the range of 2.76–8.31 μg.g^−1^ f.w. for resistant varieties.

## 3. Experimental

### 3.1. Standards

Both *trans*-resveratrol and *trans*-piceid were purchased from Sigma-Aldrich (Prague, Czech Republic).

### 3.2. Sample Preparation

Using a Open FluorCam imaging fluorometer (P.S. Instruments, Ltd., Brno, Czech Republic), the distribution parameter Φ_II_ was measured. Red wine varieties Cerason and Laurot originated from interspecific crosses ‘Merlan’ (‘Merlot’ × Seibel 13 666) × ‘Fratava’ (Blaufränkisch × St. Laurent); white wine variety Malverina originated from crosses Rakish (Villard blanc × Frühroter Veltliner) × Merlan (Merlot × Seibel 13 666) and Hibernal originated from crosses Chancellor × Riesling Weiss.

For the analysis of phenolic compounds (especially the two isomers of resveratrol) in leaves, three samples (the tissue in the center of the infection, the tissue 1 cm distant from the infection, and healthy tissue) from each measured leaf were taken as cuts of circular shape using a cork borer. Diameter of the cuts was 14 mm and the area was 154 mm^2^. Samples were weighed, placed in vials, and 0.5 mL of 70% methanol was added. Extraction was for 24 h at room temperature in darkness, with occasional shaking. The methanolic extract was then collected, passed through a glass filter, and analyzed using HPLC. After extraction, the cut leaf samples were lyophilized and dry cuts were extracted using ethyl acetate. Ethyl acetate was then evaporated with nitrogen flow and the residue dissolved in 0.5 mL of methanol. Extracts were stored at −20 °C until HPLC measurement. Both extracts were analysed separately.

### 3.3. Photo Isomerization Experiments

The solution of *trans-*piceid in a 4 mL open vial in 50% methanol was irradiated by UV lamp (Camag, Switzerland, Catalog No. 29010, Serial No. 921022) at 254 nm and laboratory temperature.

### 3.4. Samples Analysis

The samples were analyzed using an HP 1050 (Ti-series) HPLC instrument (Hewlett Packard, Palo Alto, CA, USA) on a 3 μm, 150 mm × 2 mm, Luna C18(2) column (Phenomenex, Torrance, CA, USA) with water-acetonitrile-*o-*phosphoric acid mobile phase. Mobile phase A used 5% of acetonitrile + 0.1% of *o*-phosphoric acid; mobile phase B used 80% of acetonitrile + 0.1% of *o*-phosphoric acid. The gradient was increased from 20% of B to 80% of B during 20 min and from 80% of B to 100% of B during 5 min. Flow rate was 0.250 mL min^−1^ and column temperature 25 °C. Injection volume was 5 μL. Also used were an HP G1315B diode array detector (DAD, Hewlett-Packard), with detection wavelengths at 220 and 315 nm, and scanning range 190–600 nm, as well as an HP G1321A fluorescence detector (FLD, Hewlett-Packard), with excitation wavelength 315 nm, emission wavelength 395 nm, and scanning of emission in the range of 300–600 nm. Finally, the method was validated in terms of linearity, limits of detection, and repeatability, and it was applied to the analysis of leaves extracts samples. Detection limits for piceid and 2,4,6-trihydroxyphenanthrene-2-*O*-glucoside were calculated, and were found to be 0.128 μg/mL for *trans-*piceid and 19.2 ng/mL for 2,4,6-trihydroxyphenanthrene-2-*O*-glucoside.

LC-MS was performed using an LCQ Accela Fleet (Thermo Fisher Scientific, San Jose, CA, USA) equipped with electro-spray (ESI), atmospheric pressure chemical (APCI) and atmospheric pressure photo (APPI) ionization sources and a photodiode array detector. A 1.9 μm, 50 mm × 2.1 mm, Hypersil Gold column (Thermo Electron Corporation, Bellefonte, PA, USA) was used with water-acetonitrile-formic acid mobile phase. Mobile phase A used 5% of acetonitrile + 0.1% of formic acid; mobile phase B used 80% of acetonitrile + 0.1% of formic acid. The gradient was increased from 15% of B in 1 min to 100% of B in 6.5 min and decreased to 15% of B in 6.51 min then held up to 10 min. Injection volume was 10 μL and flow rate 0.400 mL min^−1^. APCI capillary temperature was 275 °C, APCI vaporizer temperature 400 °C, sheath gas flow 58 L min^−1^, auxiliary gas flow 10 L min^−1^, source voltage 6 kV, source current 5 μA, and capillary voltage 10 V.

## 4. Conclusions

This work demonstrates identification of highly fluorescent compounds in vine leaves infected by *Plasmopara viticola* using reversed phase liquid chromatography with simultaneous diode array and fluorometric detection. 2,4,6-Trihydroxyphenanthrene-2-*O*-glucoside was identified for the first time as a main fluorescent natural product arising in relation to *Plasmopara viticola* impact on the vine leaves*.* The identification was performed by line spectral comparison with a standard obtained by UV photo isomerization of *trans-*resveratrol glucoside and its structure was confirmed by liquid chromatography-mass spectrometry.
